# Facile Preparation of Stereoblock PLA From Ring-Opening Polymerization of *rac*-Lactide by a Synergetic Binary Catalytic System Containing Ureas and Alkoxides

**DOI:** 10.3389/fchem.2018.00547

**Published:** 2018-11-09

**Authors:** Ze Kan, Wenlong Luo, Tong Shi, Chuanzhi Wei, Binghao Han, Dejuan Zheng, Shaofeng Liu

**Affiliations:** Key Laboratory of Biobased Polymer Materials, Shandong Provincial Education Department, College of Polymer Science and Engineering, Qingdao University of Science and Technology, Qingdao, China

**Keywords:** homogeneous catalysis, ring-opening polymerization, stereoselective catalysis, biodegradable polyester, rac-lactide

## Abstract

Ring-opening polymerization (ROP) of cyclic esters/lactones by efficient catalysts is a powerful method for synthesis of biodegradable and biocompatible polyesters with well-defined structures. To develop catalytic systems that are fast, selective and controlled is a persistent effort of chemists. In this contribution, we report a binary urea/alkoxide catalytic system that could catalyze ROP of *rac*-LA in a fast (over 90% conversion within 1–2 min), stereoselective (*P*_i_ up to 0.93) and controlled manner, indicated by narrow MW distributions, linear relationship between the monomer conversions and *M*_n_s, end-group fidelity, and chain extension experiments. Remarkably, the catalytic system described here is simple, easily prepared, and structurally tunable and thus has versatile catalytic performances.

## Introduction

Polylactide (PLA) is one of the most popular and commercial polymers in our daily lives (Dechy-Cabaret et al., 2004; Rasal et al., 2010; Raquez et al., 2013). For one thing, it is derived from renewable resources and could completely degrade to metabolites by hydrolysis after several months of exposure to moisture,(Castro-Aguirre et al., 2016; Farah et al., 2016) which could immensely reduce consumption volume of fossil resources and environmental pollution and makes it being a perfect substituent for petroleum-based plastics (Auras et al., 2004; Ray and Bousmina, 2005). On the other hand, it has excellent biocompatibility and wide applications as biomedical and pharmaceutical material (Kim et al., 2003; Armentano et al., 2010; Oh, 2011). The physical and chemical properties of PLA and thus the potential applications are highly dependent on the tacticity of PLA chains (Thomas, 2010). The stereoregular PLA generally has higher degree of crystallinity, melting temperature and mechanical strength as compared to the atactic polymer. For example, atactic PLA is an amorphous material, while heterotactic PLA is a semicrystalline polymer having a low *T*_m_ around 120°C. In contrast, isotactic PLLA is a crystalline material with a *T*_m_ of 170°C and high mechanical strength, which make it practical useful in the area of degradable packaging, 3D printing and surgical implants (Auras et al., 2004; Guo et al., 2015). Furthermore, the stereocomplex of *L*-PLA and *D*-PLA in 1:1 ratio (Ikada et al., 1987; Tashiro et al., 2017) or stereoblock PLA prepared from *rac*-LA using stereoselective catalysts (Stanford and Dove, 2010; Thomas, 2010; Dijkstra et al., 2011) has an even higher *T*_m_ up to 230°C and improved mechanical properties, due to cocrystallization of enantiomers (Ovitt and Coates, 2000; Fujita et al., 2008).

In the past decades, many new catalysts or initiators, such as enzymatic (Duskunkorur et al., 2015), metal-based (O'Keefe et al., 2001; Jensen et al., 2004; Arbaoui et al., 2008; Arbaoui and Redshaw, 2010; Carpentier, 2010; Sauer et al., 2013; Mou et al., 2014; Walton et al., 2014; Liu et al., 2017; Luo et al., 2017; Wang et al., 2017; Xu et al., 2017; Shi et al., 2018a,b) and organic catalysts (Kamber et al., 2007; Song et al., 2017), have been reported for conventional ROP of LA (Dechy-Cabaret et al., 2004). However, there are few able to prepare stereoregular PLA from a feedstock of *rac*-LA.(Stanford and Dove, 2010; Thomas, 2010; Dijkstra et al., 2011) Salen-Al metal complexes and the derivatives reported by Spassky et al. (1996) and others (Radano et al., 2000; Nomura et al., 2002, 2007; Ovitt and Coates, 2002; Zhong et al., 2002, 2003; Majerska and Duda, 2004; Hormnirun et al., 2006; Du et al., 2007; Luo et al., 2017) have attracted the most attention in the last two decades. Correspondingly, two distinguishing mechanisms, enantiomorphic site control mechanism (Ovitt and Coates, 2002) and chain end control mechanism (Nomura et al., 2002), have been proposed for the production of isotactic polymer (PLLA or PDLA) and stereoblock PLA, respectively. Concerning metal free products or processes desired, ROP of LA using organocatalysts have attracted considerable attention (Kamber et al., 2007; Kiesewetter et al., 2010). Among these, *N*-heterocyclic carbenes (NHCs) (Dove et al., 2006), phosphorics acids (Makiguchi et al., 2014), phosphazenes (Zhang et al., 2007; Liu et al., 2018), amino acids (Sanchez-Sanchez et al., 2017), and others (Zhu and Chen, 2015) are particularly useful for stereoselective ROP of *rac*-LA.

Although the degree of stereoselectivity (*P*_i_) can easily exceed 0.9, there are still several problems for either metal-based or organic catalysts: (1) they are suffering from low catalytic rates and need hours or even days to achieve high conversions (Hormnirun et al., 2006); (2) their synthesis are tedious and complicated and thus result in high cost (Sanchez-Sanchez et al., 2017); (3) their structures are hard to modify and tune the catalytic properties (Zhang et al., 2007); (4) conditions of ROP for these systems are usually rigorous (low or high temperature) (Dove et al., 2006; Zhang et al., 2007). Therefore, many scientists have devoted to solving these issues by developing new catalytic systems, of which, however, very few can simultaneously have high activity, selectivity, and easy preparation (Zhang et al., 2016; Lin and Waymouth, 2017).

**Scheme 1:**
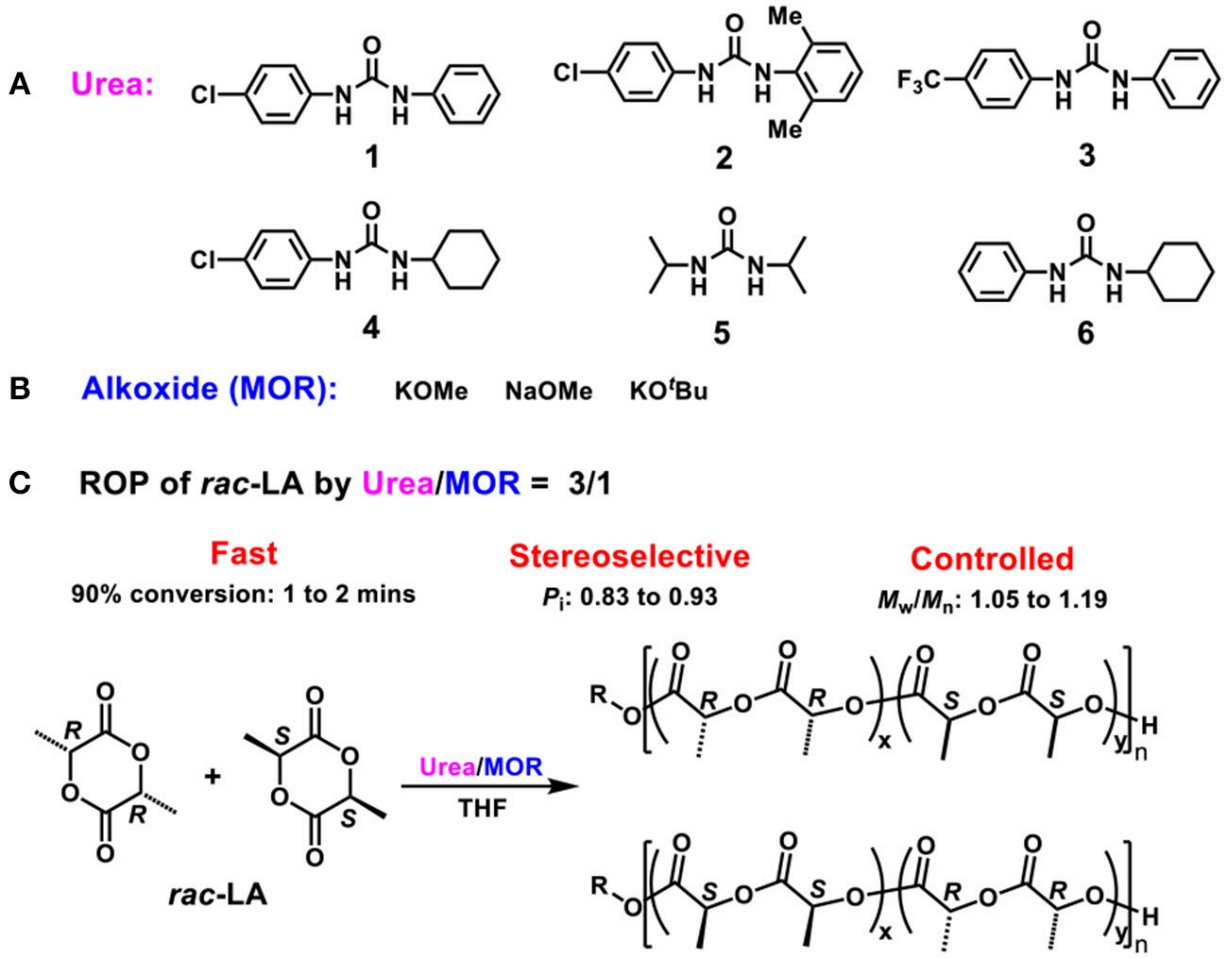
**(A)** Ureas **1–6**; **(B)** Alkoxides MOR; **(C)** ROP of *rac*-LA by binary Scheme 2 Urea/MOR catalytic system.

Catalysis employing H-bonding for initiator or monomer activation is an effective and versatile strategy in ROP (Thomas and Bibal, 2014), but stereoselective ROP of a chiral monomer using this methodology is still in the infancy. For example, β-isocupreidine reported by Chen and benzyl bispidine/thiourea reported by Dove, containing chiral centers, are efficient bifunctional catalysts for stereocontrolled ROP of *rac*-LA through the kinetic resolution, and thus produce isotactic-enriched PLLA (Miyake and Chen, 2011; Todd et al., 2013). So far, the examples of bifunctional catalysis employing H-bonding strategy that could prepare stereoblock PLA from *rac*-LA are rare and limited. Recently, Waymouth reported a binary catalytic system containing alkali metal alkoxides and organic ureas (Lin and Waymouth, 2017), which was hyperactive for ROP of different classes of cyclic monomers at room temperature, but no research on stereocontrol performances was reported. Herein, we reported that this binary urea/alkoxide system could stereoselectively catalyze ROP of *rac*-LA to produce stereoblock PLA for the first time with a superfast rate and excellent controlled manner (Scheme 1) under varying conditions.

## Results and discussion

**Figure 1:**
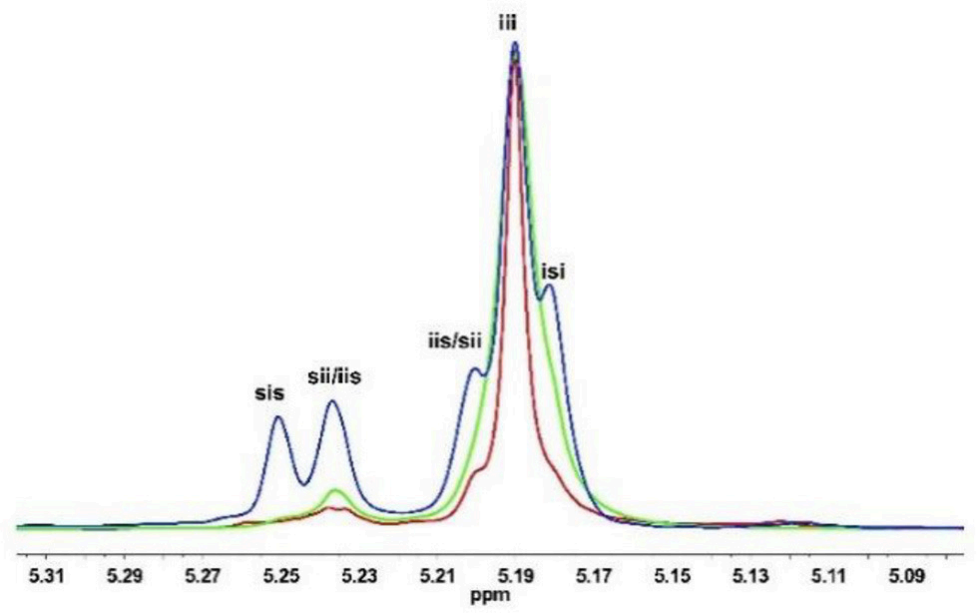
Homonuclear decoupled ^1^H NMR spectra (500 MHz, CDCl_3_) of the methine region of poly(*rac*-LA)s prepared using KOMe at 20°C (blue); Urea **1**/KOMe = 3/1 at 20°C (green); Urea **1**/KOMe = 3/1 at −60°C (red); Table 1, runs 1, 3, and 7.

At a first test, solo KOMe was used for ROP of *rac*-LA at room temperature (Table 1, run 1). The polymerization was slow and a conversion of 67% was obtained within 30 min. As expected, the produced PLA was atactic polymer (*P*_i_ = 0.52, Figure 1, blue) with a broad molecular weight distribution (Ð = *M*_w_/*M*_n_ = 1.46), which was consistent with previous report for KOMe catalyzed ROP (Zhang et al., 2016).

**Table 1:** Ring-opening polymerization of *rac*-LA by Urea/alkoxide system^*a*^.

**Run**	**Urea**	**Initiator**	**[U]_0_/[I]_0_**	**Temp.[°C]**	**Time [min]**	**Conv.*^*b*^*[%]**	***M*_n,theor_*^*c*^* [kDa]**	***M*_n,GPC_*^*d*^*[kDa]**	**Ð*^*d*^***	***P*_i_*^*e*^***
1	–	KOMe	–	20	30	67	9.7	10.8	1.46	0.52
2	1	KOMe	1	20	1	68	9.8	10.7	1.29	0.62
3	1	KOMe	3	20	1	90	13.0	13.5	1.14	0.87
4	1	KOMe	5	20	3	96	13.8	10.6	1.19	0.85
5	1	KOMe	3	−20	1	91	13.2	10.6	1.13	0.90
6	1	KOMe	3	−40	1.2	91	13.2	11.5	1.10	0.91
7	1	KOMe	3	−60	2	93	13.5	13.9	1.09	0.93
8	1	KOMe	3	−60	0.5	37	5.4	6.0	1.06	0.90
9	1	KOMe	3	−60	1	51	7.4	7.8	1.11	0.91
10	1	KOMe	3	−60	1.5	68	9.9	10.2	1.07	0.92
11*^*f*^*	1	KOMe	3	−60	2 + 3	93 + 95	13.5, 27.1	13.9, 27.6	1.06	0.92
12	1	NaOMe	3	−60	2	85	12.3	12.7	1.04	0.83
13	1	KOtBu	3	−60	1	90	13.0	13.0	1.09	0.91
14	2	KOMe	3	−60	2	80	11.5	11.8	1.10	0.91
15	3	KOMe	3	−60	2	83	11.9	11.3	1.05	0.90
16	4	KOMe	3	−60	2	96	13.8	14.0	1.10	0.91
17	5	KOMe	3	−60	240	65	9.5	10.2	1.15	0.91
18	6	KOMe	3	−60	2	90	13.0	11.8	1.10	0.91
19	–	KOMe	–	−60	60	8	–	–	–	–

a Conditions: [monomer]_0_/[Initiator]_0_ = 100/1; [monomer]_0_ = 0.2 M in THF.

b Determined by ^1^H NMR.

c M_n,theor_ = M_LA_ (144.13 g·mol^−1^) × ([LA]_0_:[I]_0_) × conversion + M_ROH_.

d Determined by GPC at 40°C in THF relative to PS standards, using a correcting factor of 0.58 (Save et al., 2002).

e Determined by homonuclear decoupled ^1^H NMR.

f *chain extension experiment*.

When 1 equiv. urea **1** was mixed with KOMe (Table 1, run 2), the resulted mixture could rapidly polymerize *rac*-LA (68% in 1 min) and produce isotactic-enriched products (*P*_i_ = 0.62). Surprisingly, when the ratio of urea to KOMe was increased to 3, superfast polymerization rate and remarkable isotacticitity were observed (90% conversion in 1 min; *P*_i_ = 0.87, Table 1 run 3, Figure 1, green). However, further increasing the quantity of urea would lead to a lower activity with similar stereoselectivity (Table 1 run 4). Therefore, next investigations were performed with a 3:1 ratio for urea/KOMe unless specified. These results are significant since the ROP of *rac*-LA with fast rate and high stereoselectivity has been achieved using a simple catalytic system under mild conditions (room temperature). In contrast, with comparative conditions (room temperature), the degrees of stereoregularity (*P*_i_) of PLA prepared by NHC and dimeric phosphazene were 0.59 and 0.72, respectively (Dove et al., 2006; Zhang et al., 2007). As compared to metal based catalysts, this urea/KOMe binary system showed comparable stereoselectivity but higher activity (Chamberlain et al., 2001; Nomura et al., 2002; Ovitt and Coates, 2002; Hormnirun et al., 2004, 2006; Bakewell et al., 2012; Pilone et al., 2014; Xiong et al., 2015).

It was reported that decreasing reaction temperature could enhance the isotacticity but at the cost of low activity (Dove et al., 2006; Zhang et al., 2007). Dimeric phosphazene with 1-pyrenebutanol as initiator produced PLAs with a *P*_i_ of 0.72 at 20°C and a *P*_i_ of 0.95 at −75°C, but the activity tremendously decreased to 1/50 (Zhang et al., 2007). Recently, we developed a new type of organic phosphazene base (**CTPB**) (Li et al., 2017; Zhao et al., 2017) and catalyzed ROP of *rac*-LA at −75°C with high stereoselectivity (*P*_i_ = 0.93) (Liu et al., 2018). In our current case, the increased isotacticity up to 0.93 was observed along with decreasing temperature (Table 1, runs 3 and 5–7). However, the polymerization rates remained fast (Figures S4–S9). At −20°C, a conversion of 91% was achieved in 1 min and the resulted PLA had a high *P*_i_ of 0.90 (Table 1 run 5). Even at −60°C, both fast polymerization rate (93% conversion in 2 min) and high *P*_i_ (0.93, Figure 1, red) were obtained (Table 1, run 7). The high isoselectivity was also confirmed by characterization of methane carbon (Figure S1, ^13^C NMR spectrum). Moreover, the *T*_m_ of the resulted PLA was 178°C (Figures S2, S10, S11) (Nomura et al., 2009), that is higher as compared to PLLA having similar MW and confirmed the formation of stereoblock PLA (Scheme 1C) (Zhang et al., 2007).

**Figure 2:**
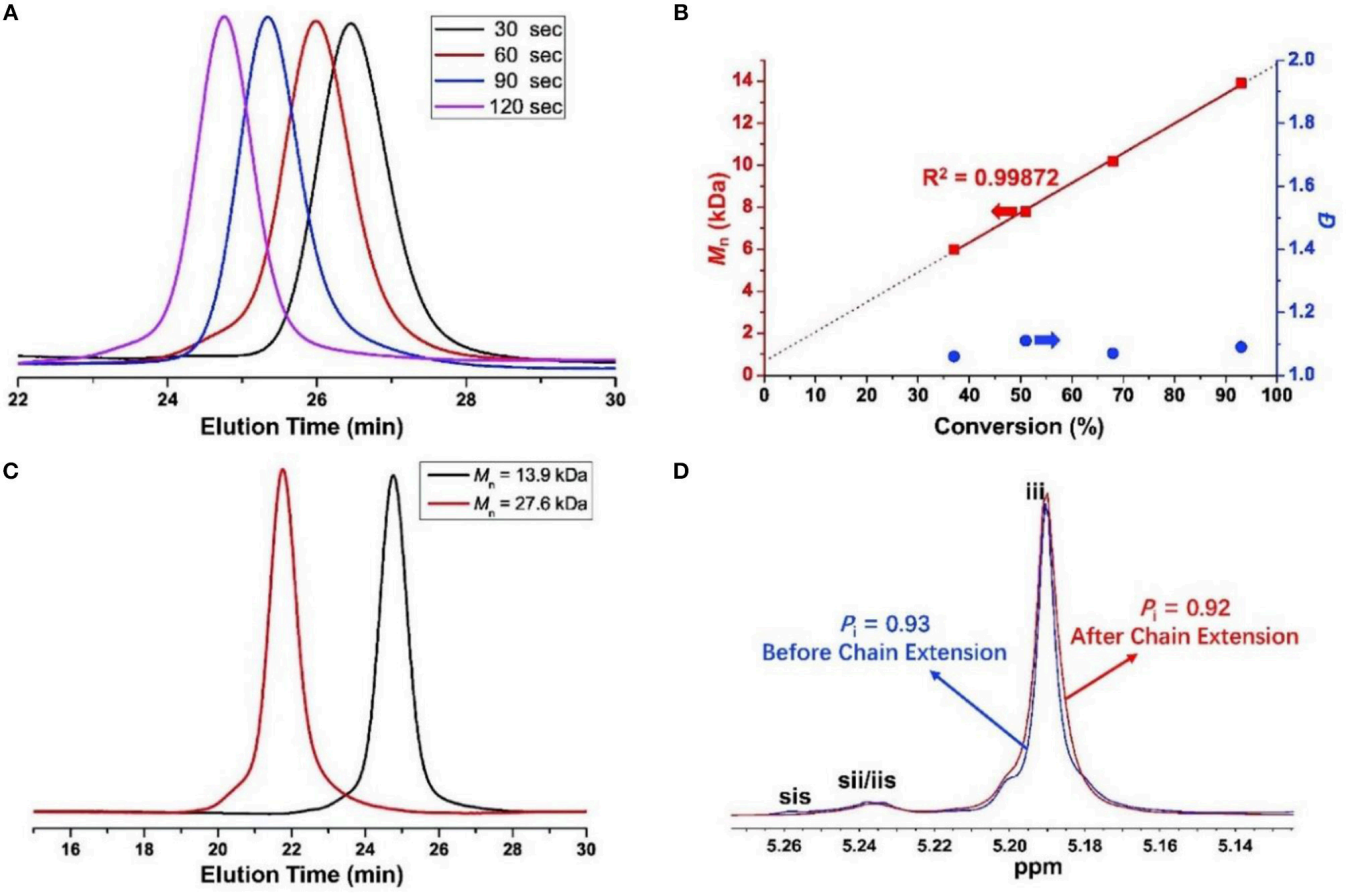
For (**A,B)**: ROP by urea **1**/KOMe in 0.5, 1, 1.5, and 2 min at −60°C (Table 1, entries 7–10), **(A)** GPC traces of PLAs; **(B)** *M*_n_ vs*. rac*-LA conversion; for **(C,D)** chain extension experiment (Table 1, entry 11), **(C)** GPC traces of PLAs (dark line, *M*_n_ = 13.9 kDa, Ð = 1.09; red line, *M*_n_ = 27.6 kDa, Ð = 1.06); **(D)** Homonuclear decoupled ^1^H NMR spectrum of the methine region of PLAs.

**Figure 3:**
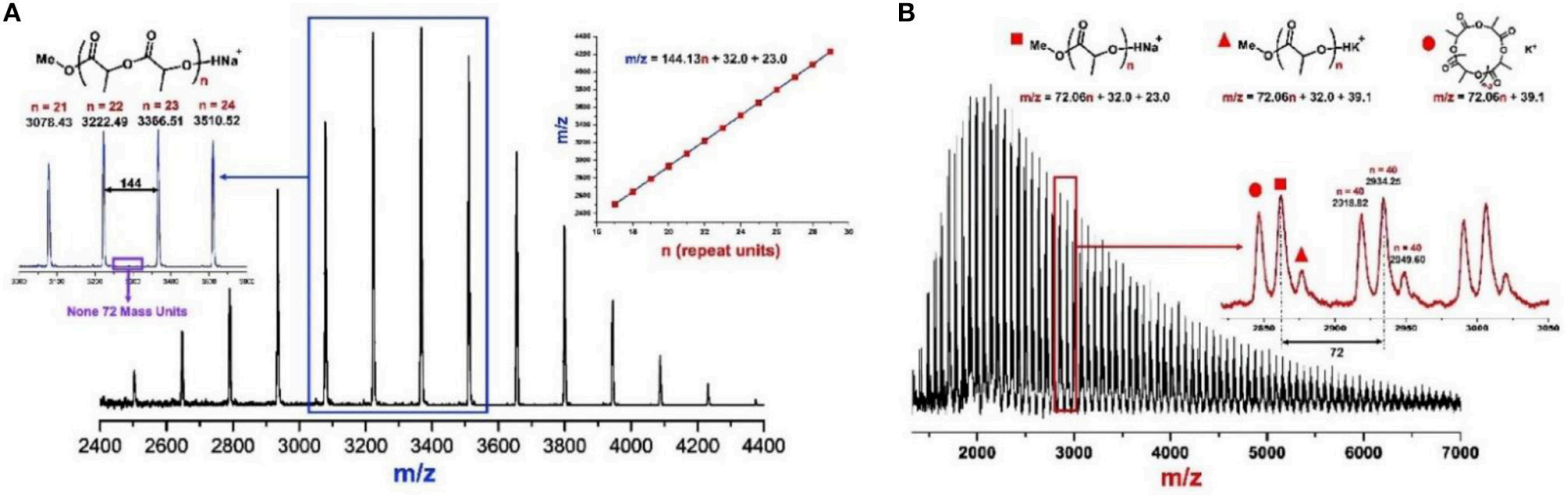
MALDI-TOF mass spectra of PLAs produced by **(A)** urea **1**/KOMe = 3/1, [M]_0_/[I]_0_ = 50, −60°C, 1 min, 85% conversion, *M*_n_ = 4.0 kDa, Ð = 1.08; and **(B)** KOMe, [M]_0_/[I]_0_ = 50, 20°C, 20 min, 70% conversion, *M*_n_ = 3.2 kDa, Ð = 1.45.

The molecular weight distributions (Ð) are narrow as shown in Figure 2A (Ð = 1.06–1.11), and the *M*_n_s of resulted PLA linearly increase with enhanced conversions of monomer as shown in Figure 2B. These results indicate that the ROP of *rac*-LA by urea **1**/KOMe at low temperature is highly controlled. The structural analysis of a low-*M*_n_ PLA was carried out with MALDI-TOF MS (Figure 3A). In Figure 3A, the ion peaks are separated by 144 mass units and consistent with linear PLA having MeO/H chain ends [*M*_n_ = 144.13 n + 32.0 (MeOH) + 23.0 (Na^+^) (g·mol^−1^)]. The transesterification reactions are negligible due to the absence of ions separated by 72 mass units, consistent with the highly controlled polymerization by urea/KOMe catalytic system. ^1^H NMR spectrum of resulted polymer (Figure S3) shows a singlet peak at 3.74 ppm, which confirms the group of O*Me* as chain end. In contrast, the MALDI-TOF MS of PLA prepared by KOMe alone exhibited three series of ions separated by 72 mass units (Figure 3B), corresponding to linear PLA having MeO/H chain end [square-series, *M*_n_ = 72.06 n + 32.0 (MeOH) + 23.0 (Na^+^) (g·mol^−1^); triangle-series, *M*_n_ = 72.06 n + 32.0 (MeOH) + 39.1 (K^+^) (g·mol^−1^)] and cyclic PLA without chain end [cycle-series, *M*_n_ = 72.06 n + 39.1 (K^+^) (g·mol^−1^)], respectively. This result indicated competitive transesterification reactions and back-biting reactions during ROP by KOMe alone.

Moreover, a chain extension experiment was carried out, in which the second 100 equiv. monomers were added into a pre-formed PLA before quenching. GPC (Figure 2C) and homonuclear decoupled ^1^H NMR analysis (Figure 2D) showed an excellent control on the *M*_n_s, distributions and stereoregulatities before (*M*_n_ = 13.9 kDa, Ð = 1.09, *P*_i_ = 0.93) and after (*M*_n_ = 27.6 kDa, Ð = 1.06, *P*_i_ = 0.92, Table 1 run 11) chain extension. This result further confirmed the highly controlled behavior of urea/KOMe system.

Besides the hyperactive, stereoselective and controlled properties, the other advantage of urea/alkoxide system is the tunable catalytic performances benefitted from the diversities of alkoxides and ureas. Replacement of the KOMe with KO^*t*^Bu led to a faster polymerization rate (90% conversion in 1 min), while the high control on molecular weight and stereoselectivity remained (Table 1 run 13). In contrast, using sodium alkoxide (NaOMe) led to a lower polymerization rate (85% conversion in 2 min) and a lower isoselectivity (*P*_i_ = 0.85, Table 1 run 12). On the other hand, a series of commercially available ureas (Scheme 1A) were studied in detail for the ROP of *rac*-LA. Generally, the ureas containing at least one phenyl group are more active than aliphatic urea under identical conditions (ureas **1–4** vs. **5**, Table 1 runs 7 and 14–17). It was found that homogeneous clear solutions were obtained by mixing ureas **1–4** and KOMe in THF, while the combination of urea **5** and KOMe led to less soluble compound, which probably resulted in low activity. According to previous report, the phenyl ring of urea could coordinate with alkali metal during the ROP, which probably increased the solubility of urea/alkoxide system (Zhang et al., 2016). The substituents on the phenyls with bulky steric hindrance and electron-withdrawing effect decreased the catalytic efficiency (ureas **2**, **3** vs. **1**), similar as previous report (Lin and Waymouth, 2017). Urea **6**, reported by Waymouth as the most active one combining with KOMe for ROP, was also investigated for comparison. Under similar conditions, it was proved to be efficient for stereocontrol ROP of *rac*-LA (*P*_i_ = 0.91; Table 1 run 18).

**Scheme 2:**
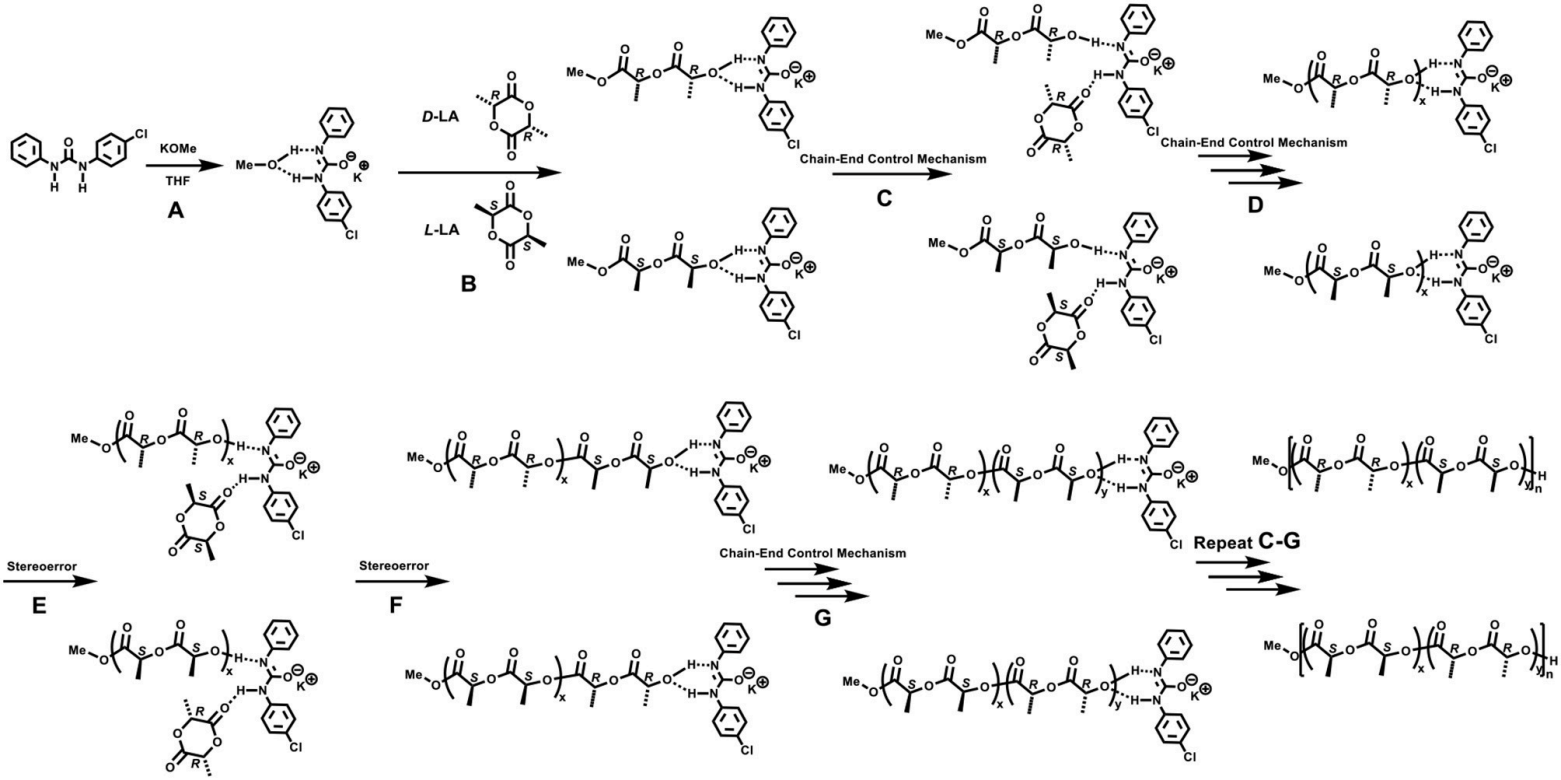
Proposed mechanism for the stereoselective ROP of *rac*-LA with urea **1**/KOMe system. **(A)** initiation process; **(B)** the first ring-opening reaction; **(C,D)** chain propagation; **(E,F)** stereoerror reaction at the chain end; **(G)** chain propagation.

Since the urea/alkoxide system had no chirality, the high stereoselectivity of ROP of *rac*-LA should be achieved through the chain end control mechanism (Table S1), similar as the previously reported one for phosphazene catalyst (Zhang et al., 2007; Liu et al., 2018). In the case of phosphazene catalyzed ROP of *rac*-LA, the bulky dimeric phosphazene and **CTPB** molecules could enhance the steric hindrances at the propagating chain end, and thus increased the stereocontrol, particularly when the mobility of the molecule was limited at low temperature. Inspired by Waymouth's recent work (Zhang et al., 2016), a synergetic bifunctional mechanism was proposed for the formation of stereoblock polymer using a feedstock of racemic monomers by achiral urea/KOMe system (Scheme 2). Reaction of the neutral urea with KOMe formed a complex of urea anion associated with activated MeOH by H-bonds (Scheme 2, A) that can initiate the ROP of *rac*-LA. In the first turnover, *D*-LA and *L*-LA monomers have an equal chance to open the ring, considering the achiral nature of urea/KOMe system. The urea anion could interact with the terminal alkoxide by H-bonds (Scheme 2B). Next, the coming monomer would interact with the N-H and insert in the polymer chain with stereocontrol because of chiral induction derived from the last inserted monomer (Schemes 2C,D). With this mechanism, *R* or *S* blocks of PLA could be constructed in the chain propagation. Occasionally, a monomer with opposite chirality was enchained and then built a new block having different configurations (Schemes 2E–G). This process (Scheme 2) was similar as that in chain end control mechanism by achiral metal based catalysts (Nomura et al., 2002). However, the existence of H-bonds (between urea anion and chain end, urea anion and the coming monomer) provided a unique bifunctional mechanism for ROP of *rac*-LA with high stereoselectivity. Note that the proposed hydrogen bonding association between urea anion and polymer chain end may also prevent back-biting forming cyclic product and transesterification reactions, which were absence in ROP by urea/KOMe system but occurred in the scenario of KOMe alone (Figure 3).

## Conclusion

The synergetic binary urea/MOR catalytic system was investigated for stereoselective ROP of *rac*-LA for the first time. At room temperature, the ROP of *rac*-LA with fast rate (90% conversion within 1 min) and high stereoselectivity (*P*_i_ = 0.87) has been achieved. At low temperature, the increased *P*_i_ up to 0.93 was obtained and the polymerization rates remained fast (over 90% conversion within 2 min). Narrow MW distributions, linear increment MW with enhanced monomer conversion and chain extension experiments suggest highly controlled ROP of *rac*-LA by urea/KOMe binary catalytic system. The microstructures and properties of the produced PLA were carefully analyzed and characterized by GPC, NMR, DSC, and MALDI-TOF. A synergetic bifunctional mechanism was proposed based on these results to elucidate the formation of stereoblock polymer using a feedstock of racemic monomers (*rac*-LA) by the binary urea/MOR catalytic system. These high-performances on catalysis together with the easy preparation and tunable structures make this binary system of great potential in practical application. Our research expands the use to H-bonding catalysis to prepare advanced well-defined polymers.

## Experimental section

### General considerations

All moisture/oxygen sensitive reactions/compounds were performed using standard Schlenk techniques or glovebox techniques in an atmosphere of high-purity nitrogen. THF, DCM, toluene was purified by first purging with dry nitrogen, followed by passing through columns of activated alumina. *rac*-LA was purchased from TCI and used without further purification. CDCl_3_ was dried over CaH_2_ and stored over activated 4 Å molecular sieves. All other chemicals were purchased from commercial suppliers and used without further purification unless otherwise noted. Reaction temperatures were controlled using an IKA temperature modulator.

NMR spectra were recorded on Bruker AV500 FT-NMR spectrometer. Matrix-assisted laser desorption/ionization time of flight mass spectrometry (MALDI-TOF MS) measurements were carried out on Bruker BIFLEX III equipped with a 337 nm nitrogen laser. α-Cyano-4-hydroxycinnamic acid was used as the matrix and sodium chloride as the cationizing agent. The GPC measurements of PLAs were collected on Agilent 1260 Infinity using THF as the eluent (flow rate: 1 mL min^−1^, at 40°C) and polystyrenes as standard. Differential scanning calorimetry (DSC) was performed using a TA differential scanning calorimeter DSC25 that was calibrated using high purity indium at a heating rate of 5°C/min. Melting temperature were determined from the second scan at a heating rate of 5°C/min following a slow cooling rate of 3°C/min to remove the influence of thermal history. *N*-(4-Chlorophenyl)-*N*′-phenylurea (**1**), *N*-(4-chlorophenyl)-*N*′-(2,6-dimethylphenyl)urea (**2**), *N*-(4-trifloromethylphenyl)-*N*′-phenylurea (**3**), *N*-(4-chlorophenyl)-*N*′-cyclohexylurea (**4**) and 1,3-diisopropylurea (**5**) were purchased from Aldrich and used as received.

### General procedure for the ROP of *rac*-LA

Polymerization of *rac*-LA using urea **1**/KOMe = 3/1 at −60°C (Table 1, run 7) was carried out as following processes. In a N_2_-filled glovebox, 288 mg of *rac*-LA (2.0 mmol) was dissolved in 8 mL of THF in a 25 mL vial containing a micro stir bar. A stock solution containing 14.3 mg of KOMe (0.2 mmol) and 148 mg of Urea **1** (0.6 mmol) in 20 mL of THF was prepared in a separate vial. The solutions were taken out of the glovebox and immersed in the cooling bath at −60°C. After equilibration at this temperature for 10 min, the polymerization was initiated by rapid addition of 2 mL of the stock solution containing the initiator and the catalyst into the *rac*-LA solution. At 2 min, the reaction was quenched by a couple drops of acetic acid. A small part of solution was taken and dried for ^1^H NMR characterization to determine the conversion. The other solution was poured into methanol (100 mL) to precipitate polymer.

### Chain extension experiment

Chain extension experiment (Table 1, run 7) was carried out as following processes. In a N_2_-filled glovebox, 288 mg of *rac*-LA (2.0 mmol) was dissolved in 8 mL of THF in a 25 mL vial containing a micro stir bar. A stock solution containing 14.3 mg of KOMe (0.2 mmol) and 148 mg of Urea **1** (0.6 mmol) in 20 mL of THF was prepared in a separate vial. The solutions were taken out of the glovebox and immersed in the cooling bath at −60°C. After equilibration at this temperature for 10 min, the polymerization was initiated by rapid addition of 2 mL of the stock solution containing the initiator and the catalyst into the *rac*-LA solution. At 2 min, a small part of solution was taken and dried for ^1^H NMR characterization to determine the conversion of the first step. Meanwhile, a pre-arranged *rac*-LA solution (288 mg, 2.0 mmol in 2 mL of THF) was added into the rest of polymerization solution at −60°C. After another 3 min, a small part of solution was taken and dried for ^1^H NMR characterization to determine the conversion of the second step. The other solution was poured into methanol (100 mL) to precipitate polymer.

## Author contributions

All authors listed have made a substantial, direct and intellectual contribution to the work, and approved it for publication.

### Conflict of interest statement

The authors declare that the research was conducted in the absence of any commercial or financial relationships that could be construed as a potential conflict of interest.
