# Supplementary material for: Facile Preparation of Stereoblock PLA From Ring-Opening Polymerization of rac-Lactide by a Synergetic Binary Catalytic System Containing Ureas and Alkoxides
Source: Front Chem. 2018 Nov 9;6:547. doi: 10.3389/fchem.2018.00547 (PMC6240762; doi:10.3389/fchem.2018.00547)
Supplement: Supplementary file 1 [file Data_Sheet_1.docx]

**Facile Preparation of Stereoblock PLA from Ring-Opening Polymerization of *rac*-Lactide by a Synergetic Binary Catalytic System Containing Ureas and Alkoxides**

Ze Kan,^#^ Wenlong Luo,^#^ Tong Shi, Chuanzhi Wei, Binghao Han, Dejuan Zheng, and Shaofeng Liu*

Key Laboratory of Biobased Polymer Materials, Shandong Provincial Education Department, College of Polymer Science and Engineering, Qingdao University of Science and Technology, Qingdao 266042, China


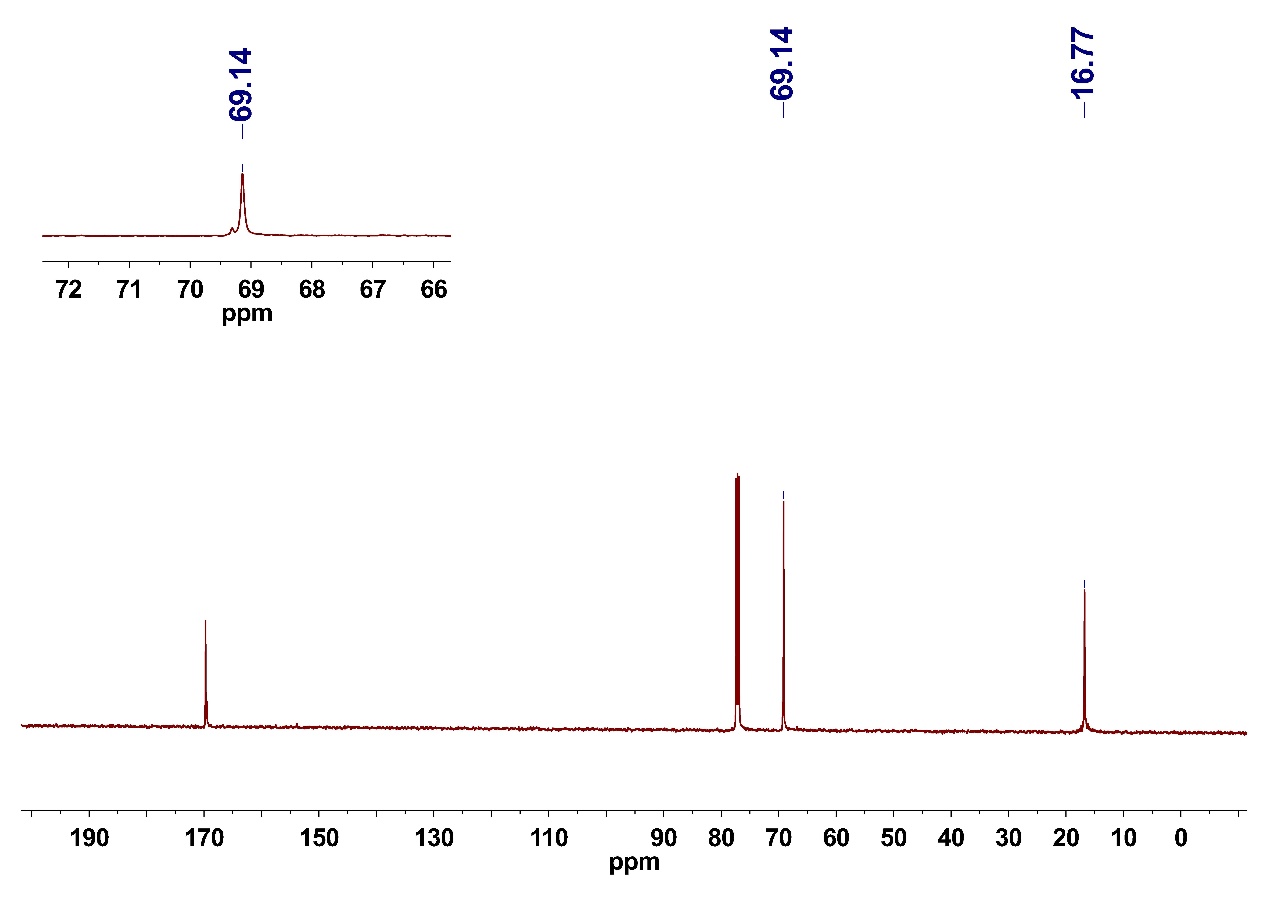


**Figure S1.** ^13^C NMR spectrum (100 MHz, CDCl_3_) of the methine region of PLA prepared using urea **1**/KOMe = 3/1 at -60 °C (Table 1, run 7).


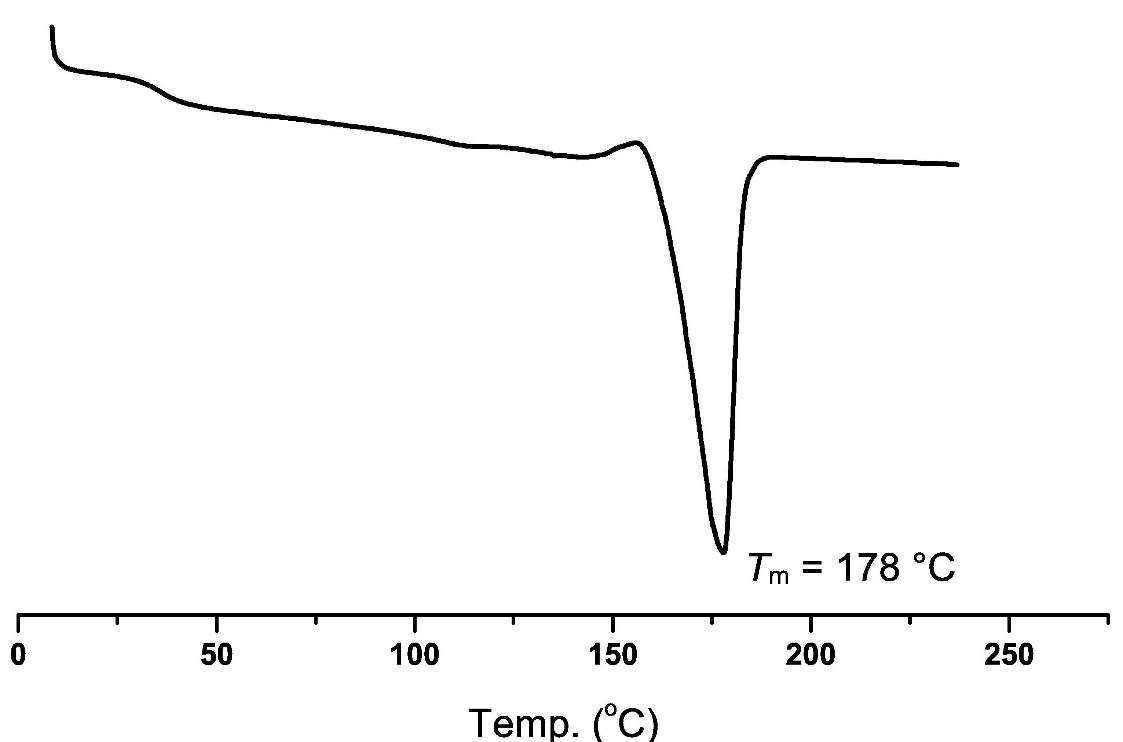


**Figure S2.** Thermal analysis (heating rate of 5 °C/min, 2^nd^ scan) of PLA prepared using urea **1**/KOMe = 1/3 at -60 °C for 2 min (Table 1 run 7).


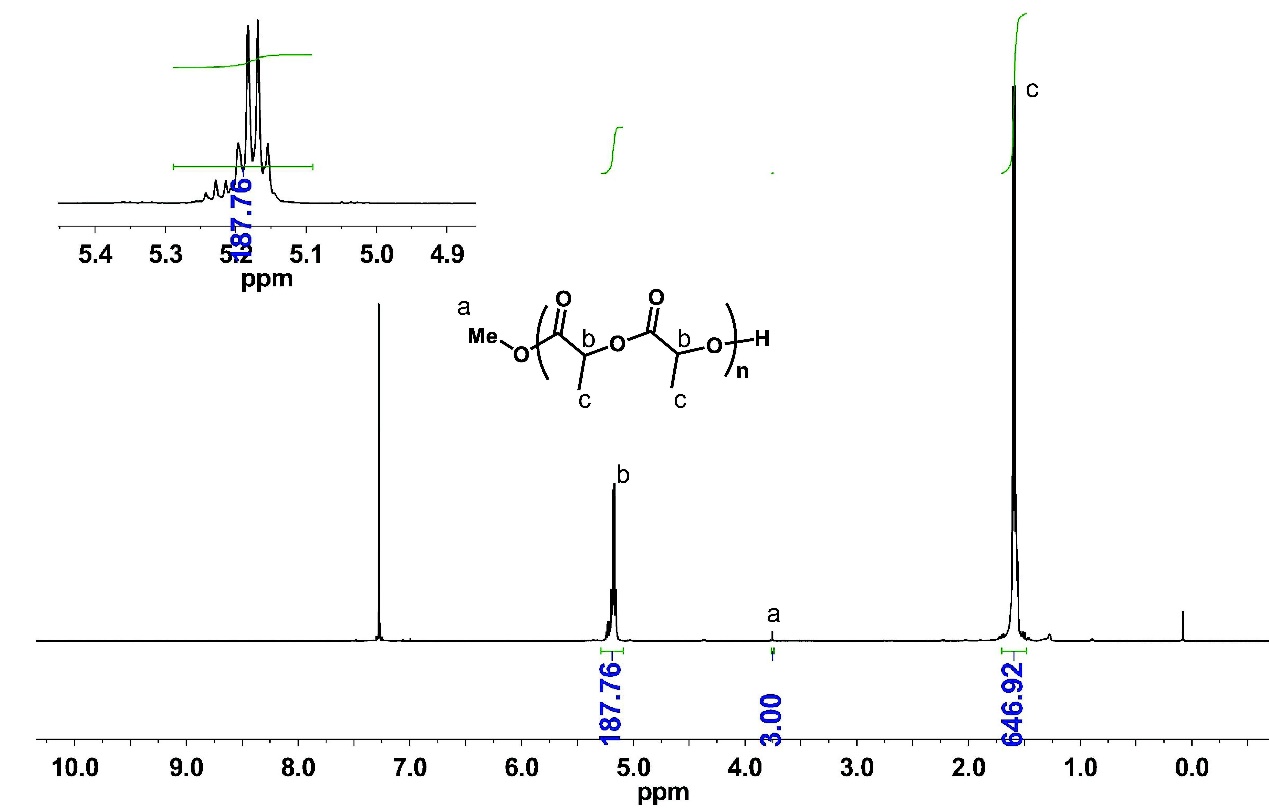


**Figure S3.** ^1^H NMR spectrum (500 MHz, CDCl_3_) of PLA prepared using urea **1**/KOMe = 1/3 at -60 °C for 2 min (Table 1, run 7).


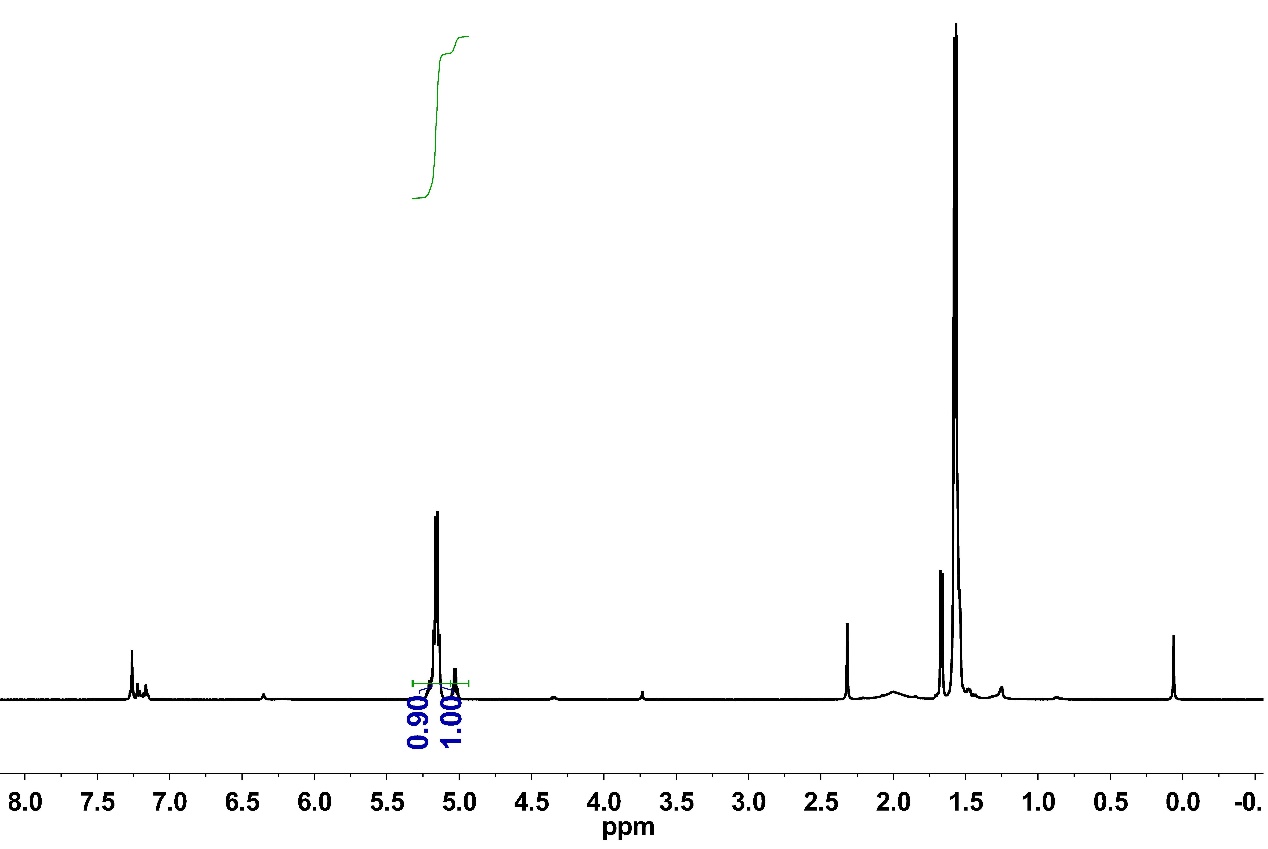


**Figure S4.** ^1^H NMR spectrum (500 MHz, CDCl_3_) of crude reaction mixture using urea **1**/KOMe = 1/3 at 20 °C for 1 min (Table 1, run 3), 90% conversion.


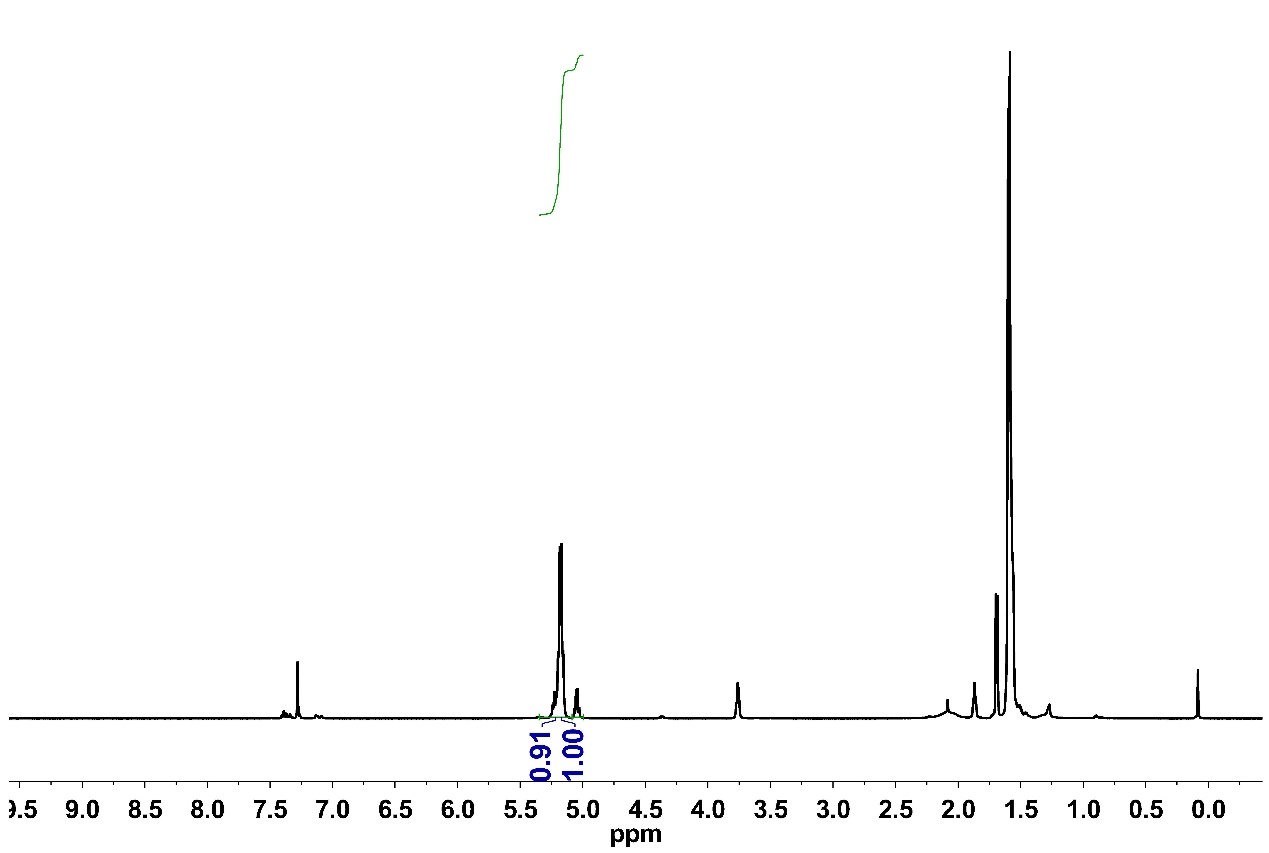


**Figure S5.** ^1^H NMR spectrum (500 MHz, CDCl_3_) of crude reaction mixture using urea **1**/KOMe = 1/3 at -20 °C for 1.0 min (Table 1, run 5), 91% conversion.


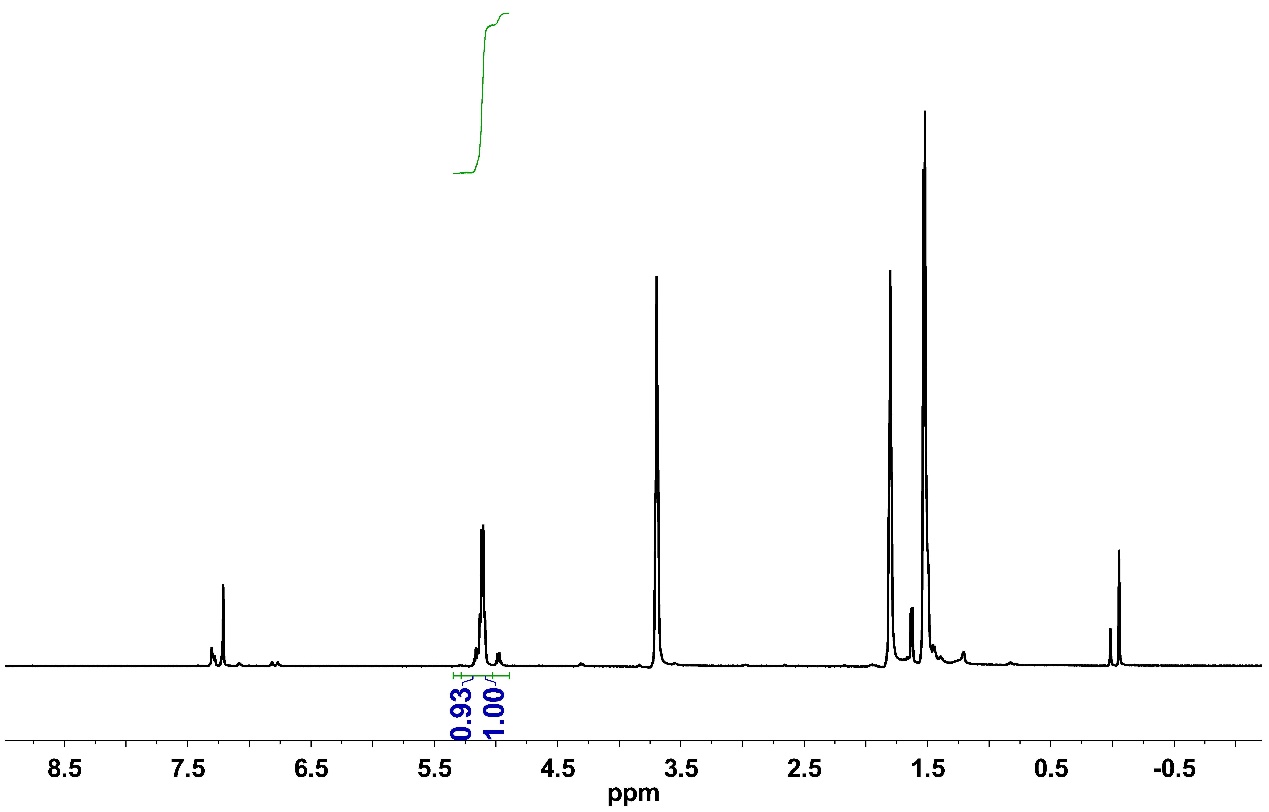


**Figure S6.** ^1^H NMR spectrum (500 MHz, CDCl_3_) of crude reaction mixture using urea **1**/KOMe = 1/3 at -60 °C for 2 min (Table 1, run 7), 93% conversion.


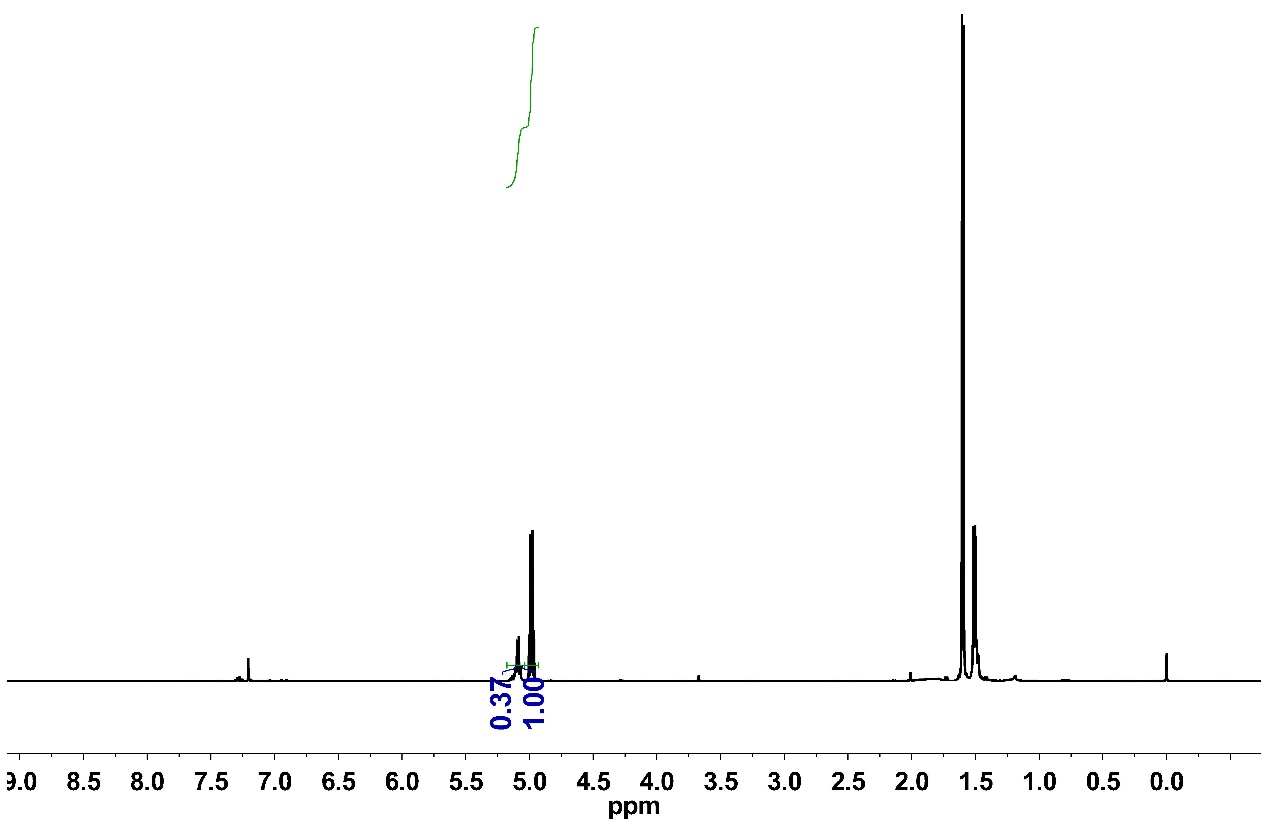


**Figure S7.** ^1^H NMR spectrum (500 MHz, CDCl_3_) of crude reaction mixture using urea **1**/KOMe = 1/3 at -60 °C for 0.5 min (Table 1, run 8), 37% conversion.


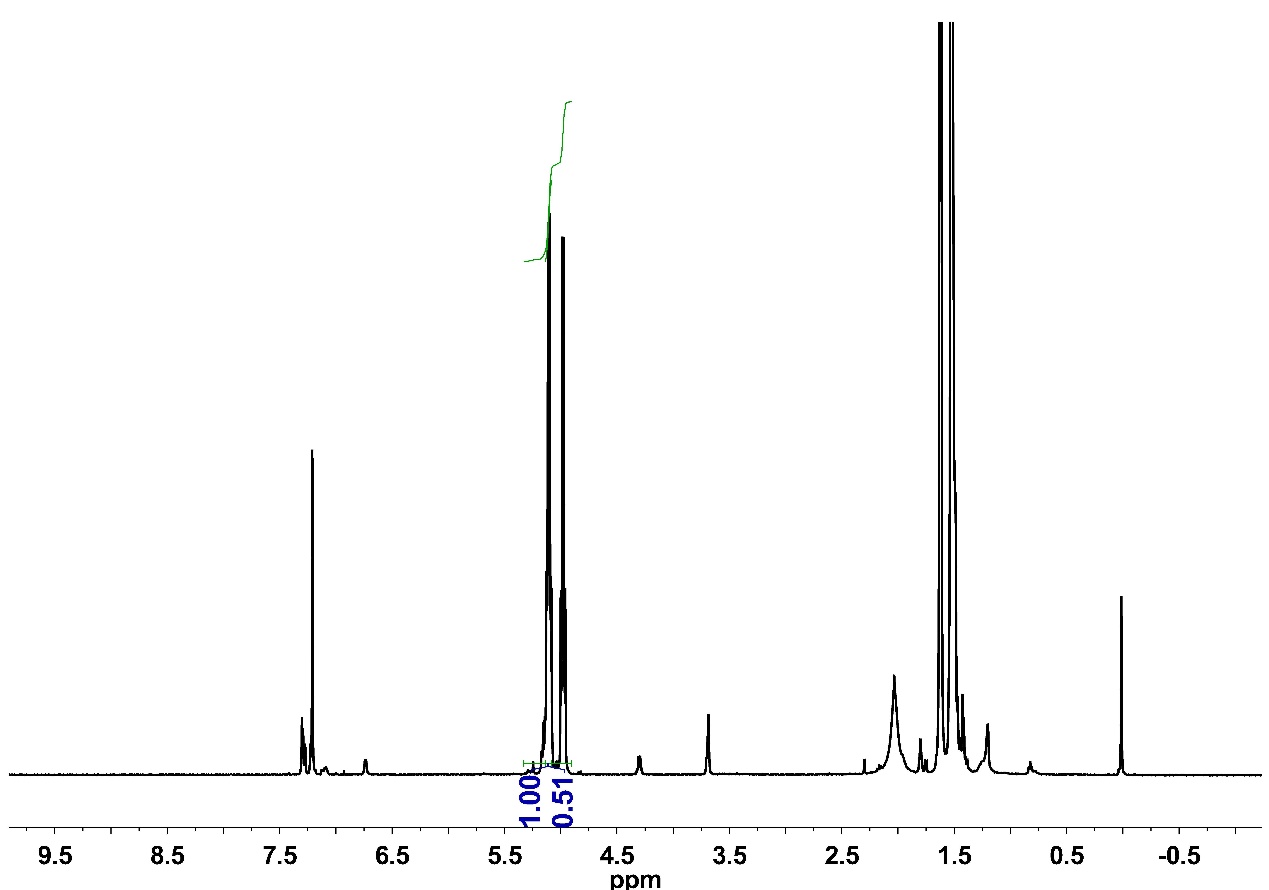


**Figure S8.** ^1^H NMR spectrum (500 MHz, CDCl_3_) of crude reaction mixture using urea **1**/KOMe = 1/3 at -60 °C for 1.0 min (Table 1, run 9), 51% conversion.


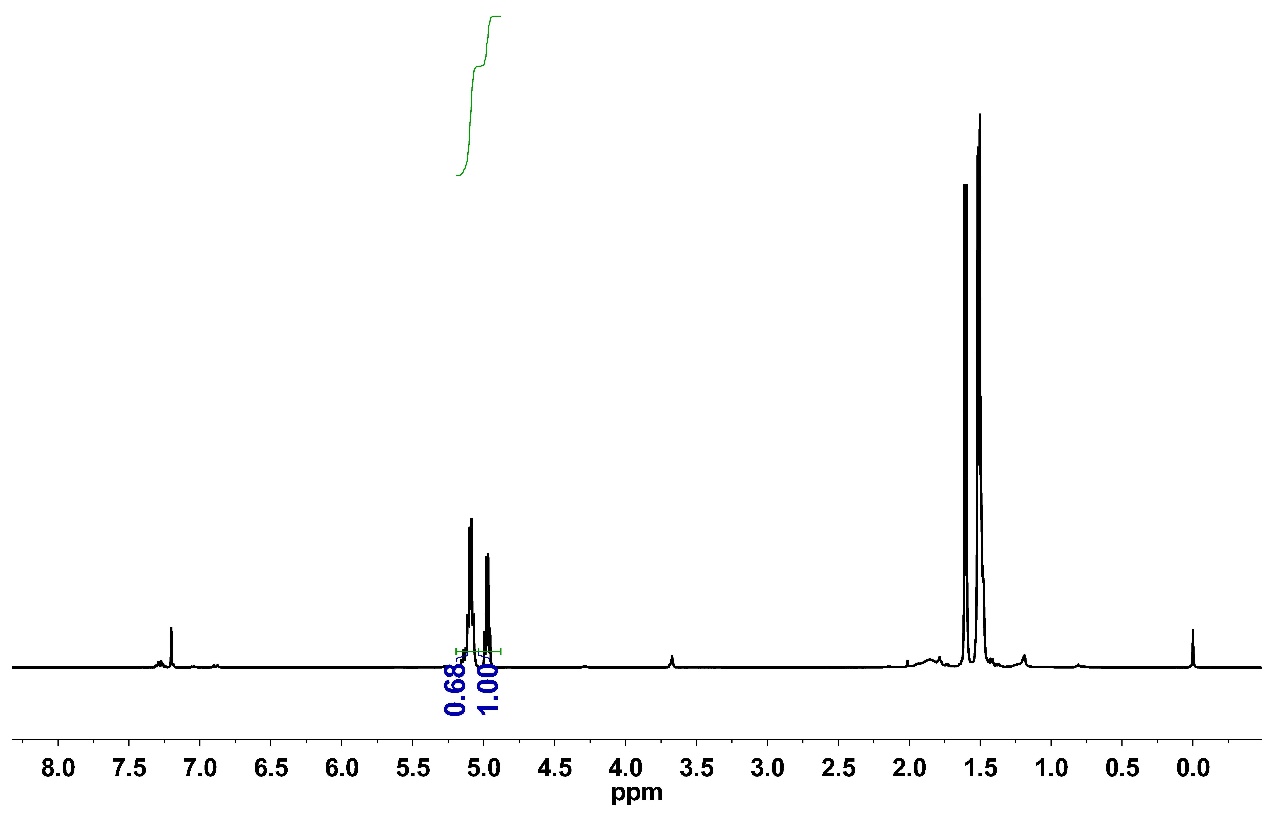


**Figure S9.** ^1^H NMR spectrum (500 MHz, CDCl_3_) of crude reaction mixture using urea **1**/KOMe = 1/3 at -60 °C for 1.5 min (Table 1, run 10), 68% conversion.


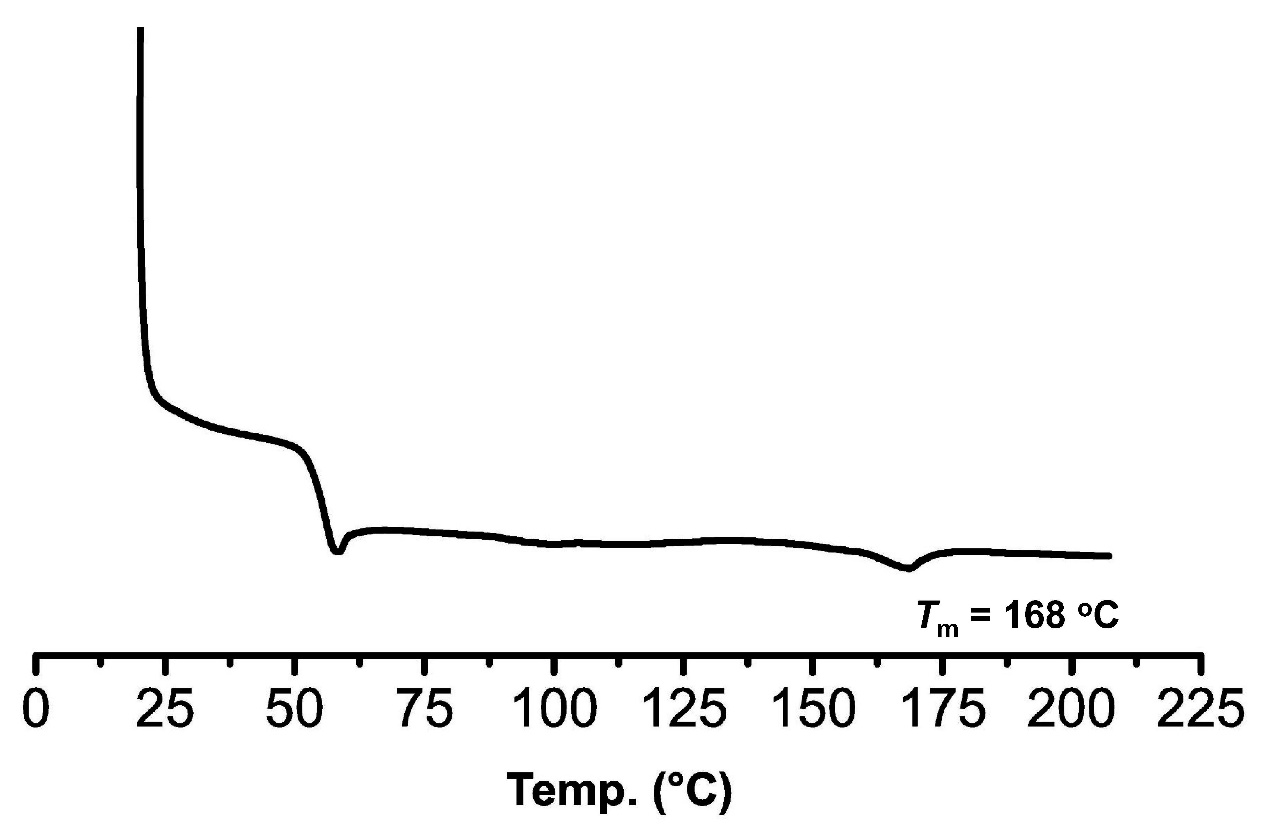


**Figure S10.** Thermal analysis (heating rate of 5 °C/min, 2^nd^ scan) of PLA prepared using urea **1**/KOMe = 1/3 at 20 °C for 1 min (Table 1 run 3).


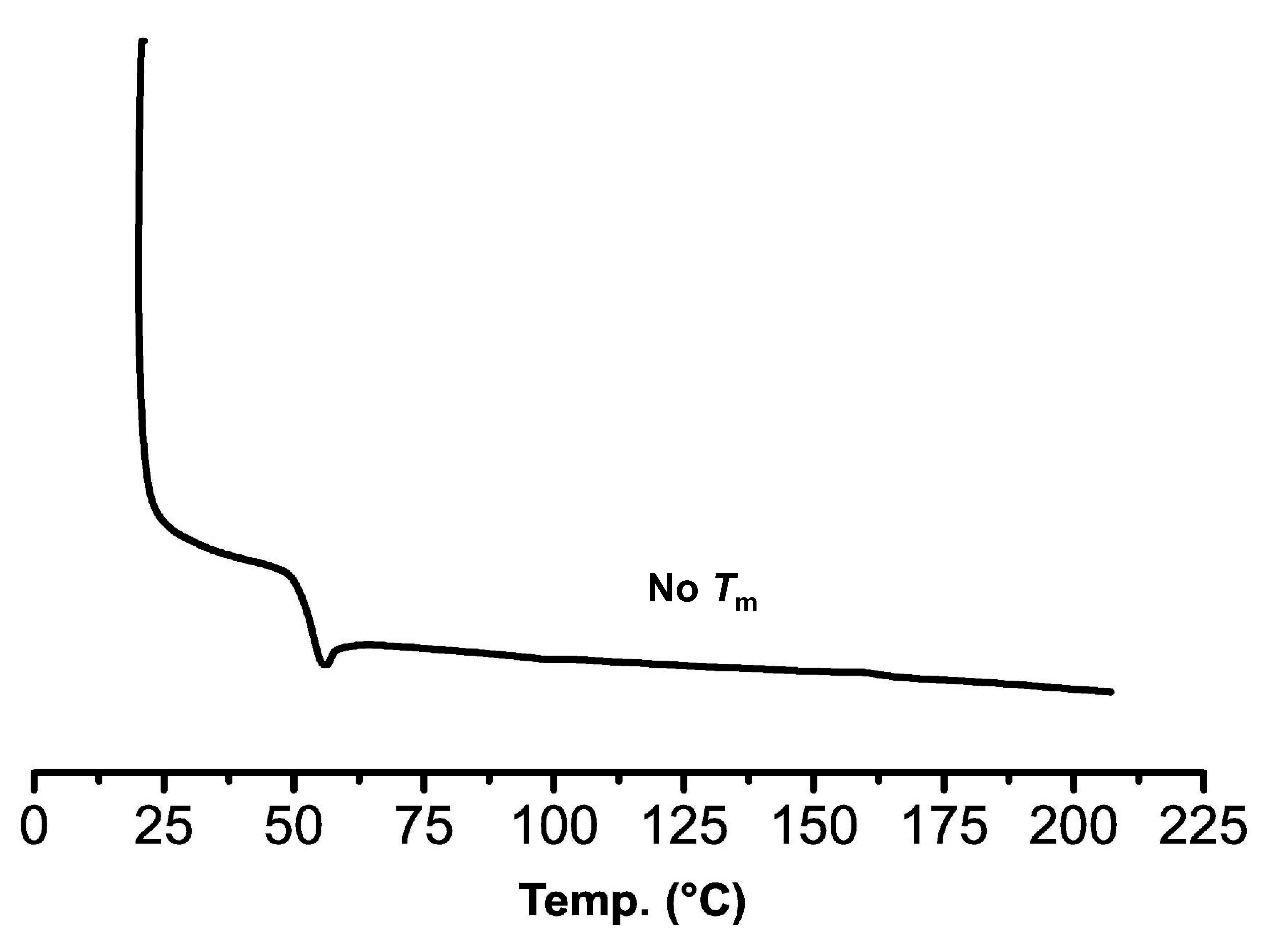


**Figure S11.** Thermal analysis (heating rate of 5 °C/min, 2^nd^ scan) of PLA prepared using urea KOMe at 20 °C for 30 min (Table 1 run 1).

**Table S1.** Ring-Opening Polymerization of *rac*-LA by Urea 1/KOMe.*^a^*

| run | Time [min] | Conv.*^b^* [%] | ee*^c^* |
| --- | --- | --- | --- |
| 1 | 0.5 | 37 | 0.0 |
| 2 | 1 | 51 | 0.0 |
| 3 | 1.5 | 68 | 0.0 |
| 4 | 2 | 93 | 0.0 |

*^a^*Conditions: [*rac*-LA]_0_/[Urea]_0_/[KOMe]_0_ = 100/3/1; [monomer]_0_ = 0.2 M in THF; -60 °C. *^b^*Determined by ^1^H NMR. *^c^*Enantiomeric excess of the unreacted monomer measured by polarimeter.
